# Soluble fms‐like tyrosine kinase‐1‐enriched exosomes suppress the growth of small cell lung cancer by inhibiting endothelial cell migration

**DOI:** 10.1111/1759-7714.13175

**Published:** 2019-08-23

**Authors:** Dexun Hao, Yanshuang Li, Gaofeng Zhao, Mingzhi Zhang

**Affiliations:** ^1^ Department of Geriatric Respiratory Ward the First Affiliated Hospital of Zhengzhou University Zhengzhou China; ^2^ Department of Anesthesiology the First Affiliated Hospital of Zhengzhou University Zhengzhou China; ^3^ Department of Thoracic Surgery the First Affiliated Hospital of Zhengzhou University Zhengzhou China; ^4^ Department of Oncology the First Affiliated Hospital of Zhengzhou University Zhengzhou China

**Keywords:** Angiogenesis, exosomes, small cell lung cancer, soluble fms‐like tyrosine kinase‐1 (sFlt‐1)

## Abstract

**Background:**

Previous studies have reported that soluble fms‐like tyrosine kinase‐1 (sFlt‐1) possesses anti‐tumor effects by inhibiting angiogenesis in many cancers. Exosomes can be engineered as delivery vehicles for transferring functional biomolecules, such as proteins, lipids, and nucleic acids (DNA, mRNA, and miRNA) to target cells to affect inflammation, apoptosis, and angiogenesis. The purpose of this study was to investigate whether exosomes can function as efficient carriers of sFlt‐1 in vitro and in vivo, to play a role in SCLC therapy.

**Methods:**

We adopted three different methods: TEM, NTA and western blot analysis to characterize the cell‐derived exosomes from NCI‐H69 SCLC cell line and normal bronchial epithelial BEAS‐2B cell line. we next explored the effects of these exosomes on HUVE cell proliferation and migration in vitro.To verify sFlt‐1‐loaded exosomes suppress the tumor growth in vivo,we established subcutaneous xenografts in nude mice using the NCI‐H69 cell line.

**Results:**

We observed that NCI‐H69‐exo significantly increased human umbilical vein endothelial cells (HUVEC) migration compared to BEAS‐2B‐exo in vitro and in vivo. sFlt‐1 protein expression was statistically higher in BEAS‐2B‐exo than NCI‐H69‐exo. sFlt‐1 protein or sFlt‐1‐enriched exosomes can inhibit the migration of HUVECs. Furthermore, sFlt‐1‐enriched exosomes exhibited higher inhibition efficacy on pro‐angiogenesis of NCI‐H69‐exo in comparison with the same concentration of sFlt‐1 protein. Intriguingly, sFlt‐1‐loaded exosomes showed marked anti‐tumor activity by inhibiting the growth of NCI‐H69 tumor xenografts. CD31 staining revealed that sFlt‐1‐loaded exosomes significantly reduced the vascular density of experimental mice. sFlt‐1‐loaded exosomes markedly induced tumor apoptosis and inhibited tumor cell proliferation in mice.

**Conclusion:**

Exosomes from a SCLC cell line contain low levels of sFlt‐1 and significantly increased the migration of HUVECs. SFlt‐1‐enriched exosomes can inhibit NCI‐H69‐exo‐induced HUVEC migration. Exosomes enriched in sFlt‐1 have the potential to be effective therapeutic agents for SCLC.

## Introduction

Small cell lung cancer (SCLC) is a lethal tumor accounting for approximately 15% of all lung malignancies, and the five‐year survival rate is less than 7%.^1^, ^2^ SCLC is typified by rapid tumor proliferation, high vascularity, frequent relapse, early metastatic dissemination, and poor prognosis. The combination chemotherapy of cisplatin (or carboplatin) and etoposide is the standard first‐line therapeutic regimen for metastatic SCLC.^3^, ^4^ Beyond that, topotecan is the only agent approved by the Food and Drug Administration (FDA) for patients with progressive or recurrent SCLC; approval is based on three phase III trials, but the efficacy of topotecan is limited.^5^, ^6^, ^7^ Therapeutic regimens for SCLC patients have remained unchanged for 30 years, resulting in the designation of SCLC as a recalcitrant cancer.^8^, ^9^ Angiogenesis is considered to be an important step in tumor growth and development of SCLC in vivo. Previous studies have shown that SCLC has greater vascularization than non‐small cell lung cancer (NSCLC).^10^, ^11^

Exosomes are nano‐sized membranous vesicles (30–150 nm) of endocytic origin released by a wide range of cell types and that enter the bloodstream.^12^, ^13^, ^14^ Exosome cargo is involved in reprogramming recipient cells, which frequently favors tumor development.^15^, ^16^, ^17^ Exosomes can be internalized by endothelial cells and affect angiogenesis.^16^, ^17^ Zhou *et al*. found that the migration of endothelial cells was significantly stimulated by breast cancer cell‐secreted exosomes.^16^ Hsu *et al*. revealed that NSCLC‐derived exosomes can enhance tumor angiogenesis.^17^ However, the role of SCLC cell line‐derived exosomes on angiogenesis remains to be elucidated.

Recently, several studies revealed that blockade of vascular endothelial growth factor (VEGF) could be of therapeutic benefit in SCLC treatment.^18^, ^19^, ^20^, ^21^ Soluble fms‐like tyrosine kinase‐1 (sFlt‐1) is a potent endogenous selective inhibitor of VEGF, and can prevent initiation of intracellular signaling by directly trapping VEGF with high affinity.^22^, ^23^, ^24^ Previous studies have reported that sFlt‐1 possesses anti‐tumor effects by inhibiting angiogenesis in many cancers.^25^, ^26^, ^27^, ^28^ In addition, several studies have found that exosomes can be engineered as delivery vehicles for transferring functional biomolecules such as proteins, lipids, and nucleic acids (DNA, mRNA, and miRNA) to target cells.^14^, ^15^, ^29^, ^30^, ^31^, ^32^

Therefore, we analyzed the effects of exosomes from SCLC cell line NCI‐H69 and normal bronchial epithelial BEAS‐2B cells on human umbilical vein endothelial cells (HUVECs) in vitro and in vivo. Additionally, we explored whether exosomes can function as efficient carriers of sFlt‐1 in vitro and in vivo, to play a role in SCLC therapy.

## Methods

### Clinical serum samples

Human samples were obtained from voluntarily consenting patients at the First Affiliated Hospital of Zhengzhou University using protocols approved by the research ethics committee. The present study was approved by the Ethics Committee of Zhengzhou University. Written informed consent was acquired from each patient.

### Cell culture

Human umbilical vein endothelial cells (HUVECs) were isolated by a standard collagenase enzyme digestion and maintained in endothelial cell medium (ECM, ScienCell, San Diego, CA, USA) supplemented with 5% fetal bovine serum (FBS), 1% penicillin/streptomycin (P/S), and 1% endothelial cell growth supplement (ECGS). The bronchial epithelial BEAS‐2B cell line was obtained from the Cell Resource Center, Shanghai Institutes for Biological Sciences (SIBS, China) and cultured in DMEM (Hyclone, USA) containing 5% FBS and 1% P/S. NCI‐H69 was purchased from the American Type Culture Collection and grown in RPMI 1640 (Hyclone, USA) supplemented with 10% FBS, 2 mM L‐glutamine, and 1% P/S. HEK293 cells were infected with lentivirus expressing Tet‐sFlt‐1 to obtain a cell line overexpressing sFlt‐1. The efficiency of overexpression was examined by Western blot analysis. The details were outlined in a recently published study.^33^ All cell lines were cultured in a humidified atmosphere containing 5% CO_2_ at 37°C.

### Isolation of exosomes

Exosomes were isolated from the cell culture medium of BEAS‐2B and NCI‐H69 cell lines. When cells reached 80%–90% confluence in the culture flask, they were washed three times in PBS and then maintained in serum‐free medium for 48 hours. Exosomes were isolated from the culture medium using the ExoQuick‐TC kit (System Bioscience, Palo Alto, CA, USA) according to the manufacturer's instructions. The culture medium was centrifuged at 3000 ***g*** for 15 minutes at 4°C to remove cells and debris, then the supernatant was transferred to a new tube without disturbing the pellet. The supernatant was mixed with ExoQuick Exosome Precipitation Solution and incubated overnight at 4°C. The mixture was centrifuged at 1500 ***g*** for 30 minutes, and the supernatant was then discarded. The tube with the exosome pellet and residual solution was centrifuged at 1500 ***g*** for five minutes, and the supernatant was discarded. The exosome pellet was resuspended in 1× PBS and stored at −80°C.

### Transmission electron microscopy

Transmission electron microscopy (TEM; Thermo Fisher Scientific, Waltham, MA, USA) was used to observe the morphology of the exosomes and 15 μL of exosome suspension was fixed on a continuous grid for one minute, and then negatively stained with 2% uranyl acetate solution for one minute. Grids were allowed to thoroughly air dry. The grids were visualized using a FEI Tecnai G2 Spirit Transmission Electron Microscope (Thermo Fisher Scientific, Waltham, USA) at an acceleration voltage of 120 kV.

### Nanoparticle tracking analysis for exosomes

The size and distribution of exosomes were measured using ZetaView (Particle Metrix, Meerbusch, Germany). The data were evaluated using the instrument software, ZetaView 8.02.28.

### Western blot analysis

The concentration of exosomal protein was quantified using the BCA Protein Assay Kit (Beyotime, Shanghai, China) according to the manufacturer's protocol. Exosomes were lysed in lysis buffer (RIPA, Beyotime, Shanghai, China). Approximately 20 μg of protein extract per well was separated by 10% SDS‐PAGE, transferred onto polyvinylidene fluoride (PVDF, Roche, Mannheim, Germany) membranes, and then blocked for two hours at room temperature in 5% bovine serum albumin (BSA). The membrane was incubated with exosome‐specific antibody: CD63 (1:1000, System Bioscience) at 4°C overnight. The membrane was then incubated for two hours at room temperature with anti‐rabbit IgG antibody (1:5000, CST). Protein band was detected with an enhanced chemiluminescent kit (ECL; Thermo Fisher Scientific, Waltham, USA) and visualized using Quantity One (Bio‐Rad, Hercules, CA, USA).

### Cell proliferation assay

Proliferation of HUVECs was measured by CCK‐8 reagent (Dojingdo Molecular Technology, Japan). HUVECs were seeded in 96‐well plates (2 × 10^3^ cells/well) with complete ECM, and then starved for 16 hours after attachment. The cells were incubated with varying concentrations (0–100 μg/mL) of NCI‐H69‐exosomes and BEAS‐2B‐exosomes for 48 hours. Then, 10 μL of CCK‐8 reagent was added to each well for one hour and the plates were analyzed at 450 nm with a microplate reader (Bio‐Rad, Hercules, CA, USA).

### Transwell migration assay

The migration ability of HUVECs was evaluated using a modified membrane system with a pore size of 8.0 μm (Costar; Corning, NY, USA). HUVECs were pretreated with serum‐free ECM for eight hours. HUVECs (1 × 10^5^ cells/well) were resuspended in the upper chamber in 200 μL of serum‐free ECM. The lower chambers were filled with 800 μL serum‐free ECM, including a 100 μg/mL concentration of NCI‐H69‐exosomes or BEAS‐2B‐exosomes, and incubated at 37°C for 16 hours. Calcein‐AM (0.2 μg/mL, Invitrogen) was then added to each lower chamber, which stained the migrated cells after incubation at 37°C for 30 minutes. The migrated cells were observed and imaged with a Nikon Eclipse Ti fluorescence microscope (Tokyo, Japan).

### Enzyme‐linked immunosorbent assay

sFlt‐1 protein levels were evaluated by enzyme‐linked immunosorbent assay (ELISA) using an immunoassay kit (Thermo Fisher Scientific, Waltham, MA, USA) according to the manufacturer's instructions. The optical density (OD) was determined using a microplate reader (Bio‐Rad, Hercules, CA, USA) at 450 nm. Results were obtained as the concentration of sFlt‐1 (ng/mL) in the samples.

### In vivo Matrigel plug assay

Equal amounts of NCI‐H69‐exosomes and BEAS‐2B‐exosomes were mixed with ice‐cold growth factor‐reduced Matrigel (350 μL, BD Biosciences), and then the mixture was subcutaneously injected into athymic nude mice (male, six‐week‐old BALB/c). After 13 days, the Matrigel plugs were harvested and fixed in 4% formaldehyde in phosphate buffer for histological analysis. Immunohistochemistry was carried out on the paraffin sections to evaluate the expression of CD31. All animal care procedures conformed with institutional guidelines.

### Animal experiment

For this part of the study, six‐week‐old female athymic nude mice were maintained in specific pathogen‐free (SPF) conditions. The animal experiments were approved by the Animal Experimental Ethics Committee of Zhengzhou University, and were carried out according to institutional guidelines. NCI‐H69 cells (5 × 10^6^) in 150 μL PBS were injected into the right flank of athymic nude mice. Seven days after the cancer cell inoculation, treatment with sFlt‐1‐abundant or control exosomes was started, and injected into the tumors five days per week. Tumor sizes were monitored every two to three days by a digital caliper. The tumor volume in each mouse was determined by measuring tumor length (l) and width (w) and using the following formula: V = lw^2^/2. After 30 days, mice were sacrificed and paraffin‐embedded tumor tissues were prepared for immunohistochemical (IHC) staining.

### Statistical analysis

All results are based on three independent experiments, and all quantitative data are expressed as the mean ± standard deviation (SD). Statistical analyses were carried out with SPSS statistical software (version 22.0; IBM Corporation, Armonk, NY). Comparisons between two groups were performed with Student's *t*‐test. A *P*‐value of <0.05 was considered statistically significant.

## Results

### Characterization of exosomes

**Figure 1:**
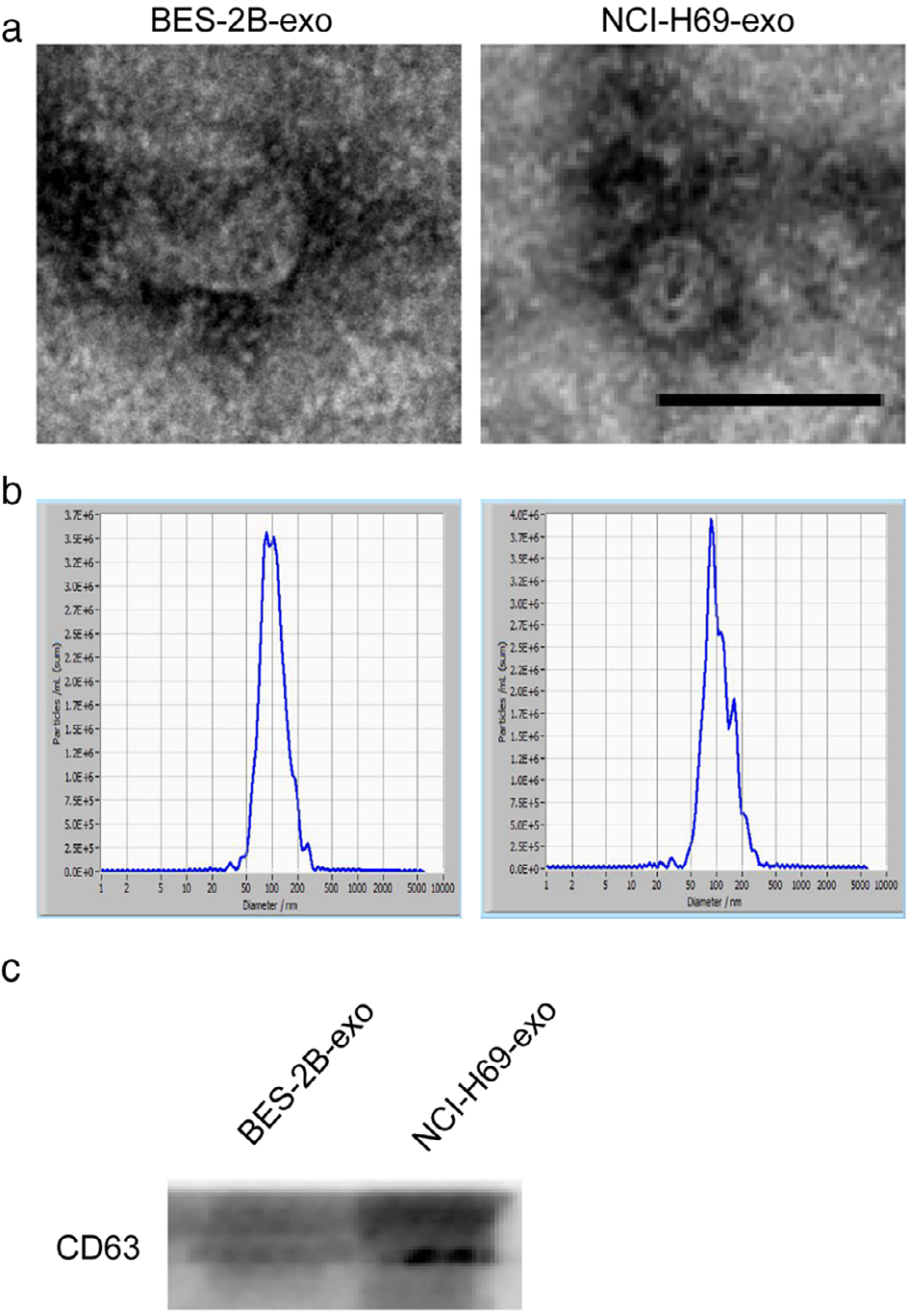
Identification of exosomes from cell lines. (**a**) Transmission electron micrographs of exosomes derived from NCI‐H69 SCLC cell line and normal bronchial epithelial BEAS‐2B cell line. Scale bar 200 nm. (**b**) The representative NTA profile of NCI‐H69‐exo and BEAS‐2B‐exo. (**c**) CD63, (common exosomes enriched marker) immunoblot of NCI‐H69‐exo and BEAS‐2B‐exo.

We chose the NCI‐H69 SCLC cell line and normal bronchial epithelial BEAS‐2B cell line as models to study the functions of cancer‐secreted exosomes. We adopted three different methods: TEM, nanoparticle tracking analysis (NTA), and western blot analysis to characterize the cell‐derived exosomes. TEM analysis and NTA, both methods revealed that NCI‐H69‐exosomes (NCI‐H69‐exo) and BEAS‐2B‐exosomes (BEAS‐2B‐exo) exhibited a typical cup‐shaped appearance and ranged in diameter from 30 to 150 nm, consistent with the characteristics of exosomes reported by previous studies (Fig 1a,b). Furthermore, the expression of an exosome‐specific marker (CD63) was verified in NCI‐H69‐exo and BEAS‐2B‐exo by Western blot analysis (Fig 1c). These results confirmed the successful isolation of exosomes from NCI‐H69 and BEAS‐2B cells.

### Exosomes derived from a SCLC cell line promote HUVEC migration

**Figure 2:**
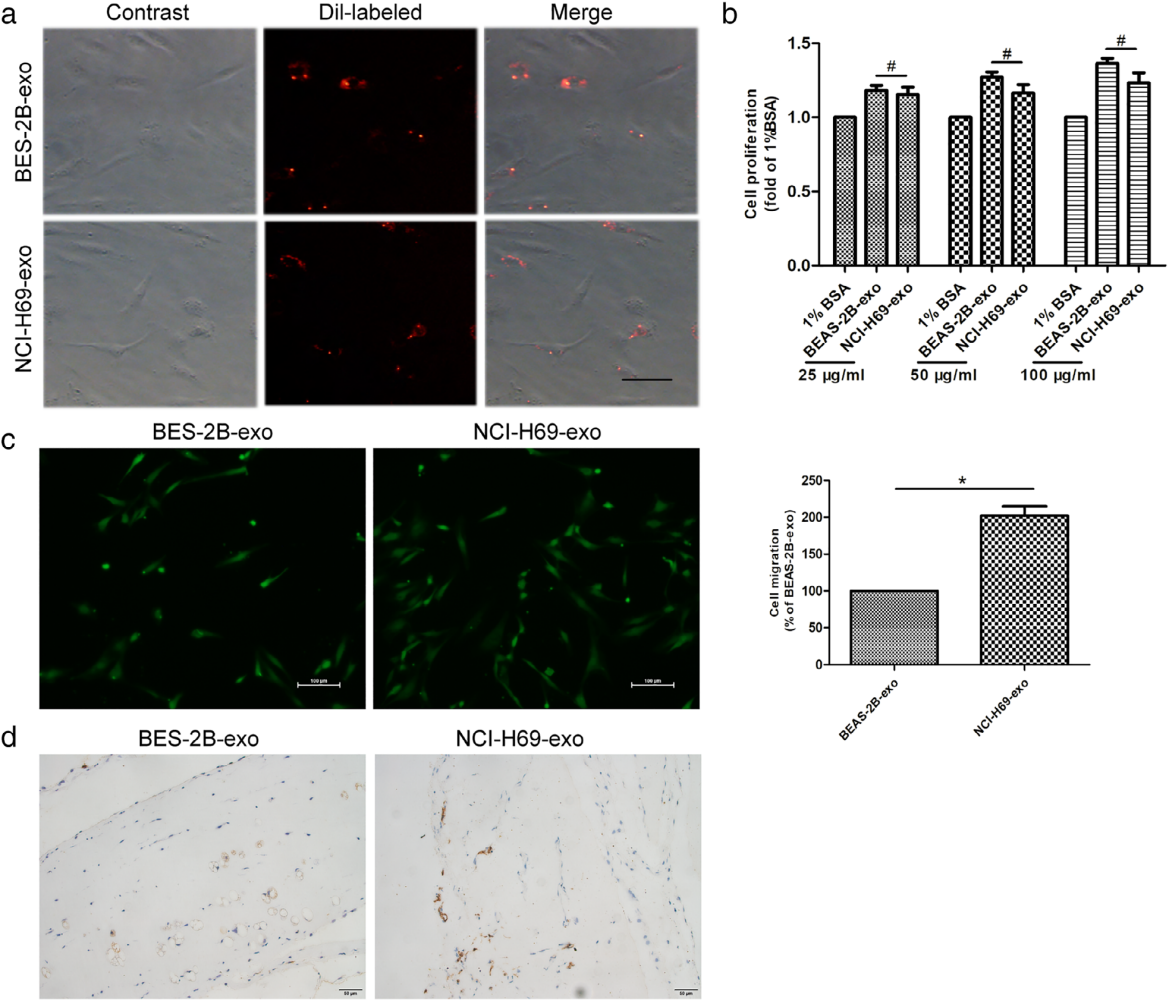
NCI‐H69‐exo induces migration of endothelial cells in vitro and in vivo. (**a**) HUVECs were incubated with Dil‐labeled exosomes (red) for 24 hours before fluorescent and phase contrast images were captured, scale bar 50 μm. (**b**) After incubation with exosomes (25, 50, 100 μg/mL), 1% BSA (negative control) for 48 hours, HUVEC proliferation was analyzed by CCK‐8 assay (NCI‐H69‐exo and BEAS‐2B‐exo, ^#^ *P* > 0.05). (**c**) Cell migration response to exosomes (100 μg/mL) was determined by transwell assay, scale bar 100 μm (*P < 0.05). (**d**) NCI‐H69‐exo increased angiogenesis in vivo, scale bar 50 μm. All Data were mean ± S.E.M. of three pairs of independent experiments.

We focused on the effects of cancer‐derived exosomes on endothelial cells, due to the critical role of endothelial cells during tumor growth and development. To investigate the biological roles of NCI‐H69‐exo and BEAS‐2B‐exo, we next explored the effects of these exosomes on HUVE cell proliferation and migration. After being incubated with Dil‐labeled exosomes for 24 hours, numerous recipient HUVECs acquired positive Dil signals as indicated by fluorescence microscopy (Fig 2a), with no statistical difference between NCI‐H69‐exo and BEAS‐2B‐exo. Figure 2b showed that there was no significant difference in HUVEC viability between NCI‐H69‐exo and BEAS‐2B‐exo incubation. Endothelial cell migration is essential for angiogenesis and tumor metastasis. NCI‐H69‐exo significantly increased HUVEC migration compared to BEAS‐2B‐exo (100 ug/mL) (Fig 2c). To explore the effect of NCI‐H69‐exo on angiogenesis in vivo, we performed a matrigel plug assay to evaluate the formation of blood vessels in athymic nude mice. Compared to BEAS‐2B‐exo, NCI‐H69‐exo significantly increased the density of neovessels in the gel plug (Fig 2d). These data show that exosomes derived from SCLC cells induced angiogenesis by enhancing the migration of HUVECs.

**Figure 3:**
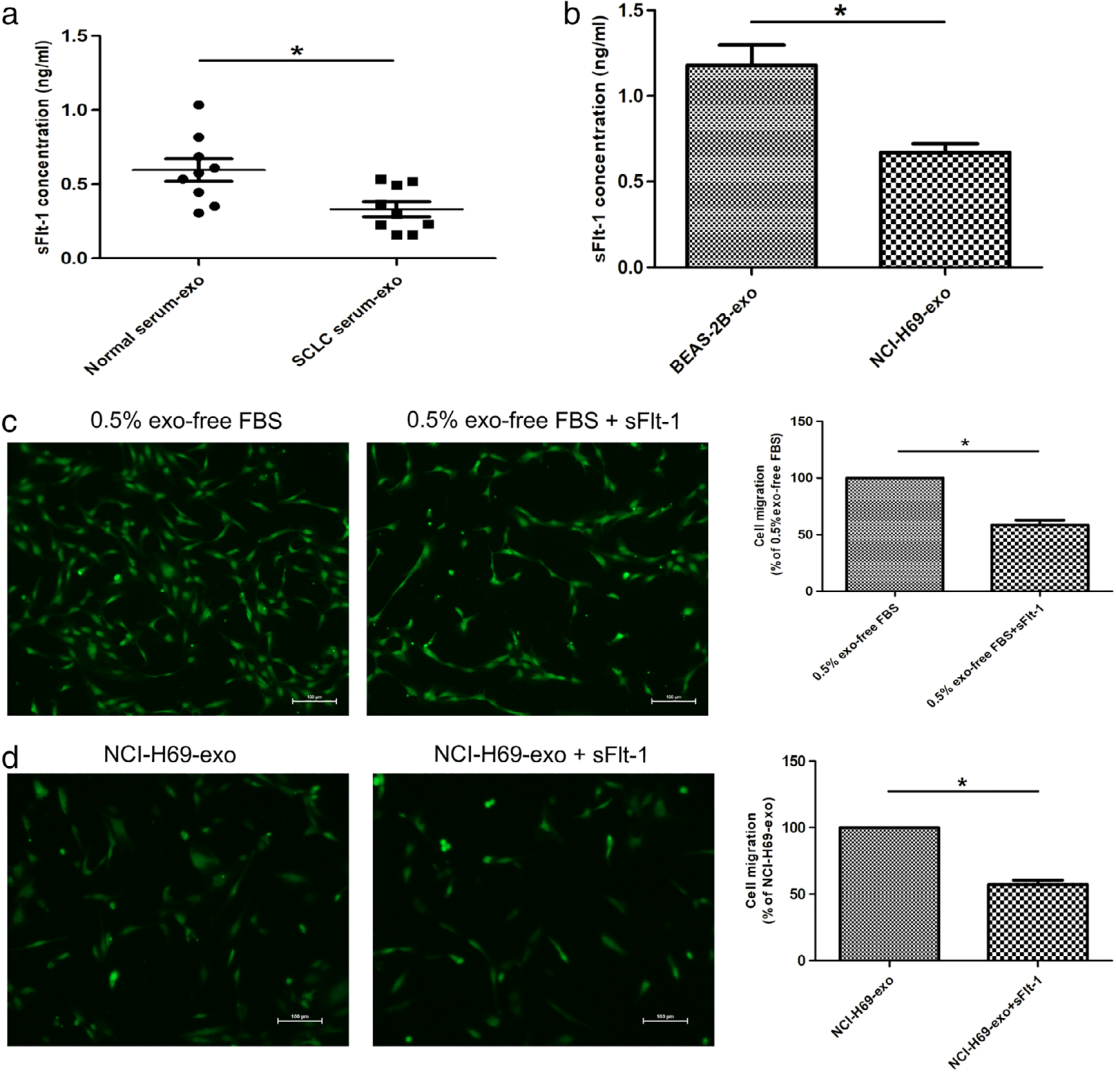
sFlt‐1 impairs HUVEC migration. (**a**) The sFlt‐1 concentrations were measured in normal serum‐exo and SCLC serum‐exo (*P < 0.05). (**b**) The sFlt‐1 concentrations were measured in BEAS‐2B‐exo and NCI‐H69‐exo (*P < 0.05). (**c**) sFlt‐1 protein (500 ng/mL) inhibited the HUVEC migration, scale bar 100 μm (*P < 0.05). (**d**) sFlt‐1 protein (500 ng/mL) reversed the proangiogenic effect of NCI‐H69‐exo (100 μg/mL), scale bar 100 μm (*P < 0.05).

Some previous studies have reported that expression of sFlt‐1 correlates significantly with a good prognosis in many tumors.^34^, ^35^, ^36^ Based on this, we measured sFlt‐1 expression in the serum‐derived exosomes of SCLC patients and healthy donors by ELISA. The level of sFlt‐1 was significantly higher in healthy donors (normal serum‐exo) compared to SCLC patients (SCLC serum‐exo) (Fig 3a). Intriguingly, we discovered that sFlt‐1 protein expression was also statistically higher in BEAS‐2B‐exo than NCI‐H69‐exo (Fig 3b). In line with the previous studies,^37^ we observed that sFlt‐1 protein obviously inhibited the migratory ability of HUVECs (Fig 3c). Meanwhile, restoring expression of sFlt‐1 abolished the angiogenesis effect of NCI‐H69‐exo on HUVECs (Fig 3d). These results suggested that decreased sFlt‐1 expression was associated with the pro‐angiogenesis of SCLC.

### SFlt‐1‐loaded exosomes inhibit HUVEC migration in vitro

**Figure 4:**
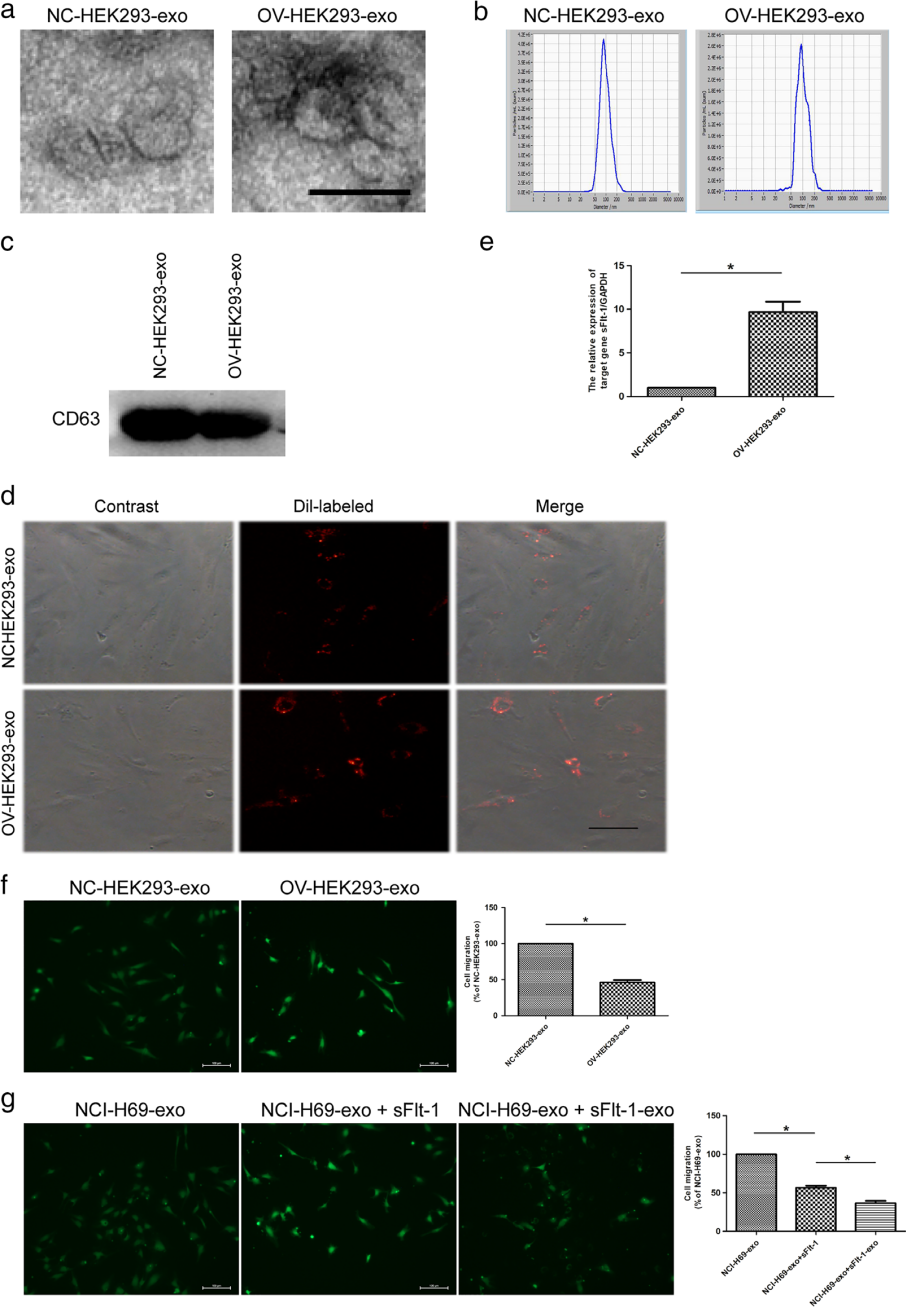
sFlt‐1‐enriched exosomes impair HUVEC migration. (**a**) Transmission electron micrographs of exosomes derived from negative control (NC) and overexpressed (OV) HEK293 cells. Scale bar 200 nm. (**b**) The representative NTA profile of NC‐HEK293‐exo and OV‐HEK293‐exo. (**c**) CD63, (common exosomes enriched marker) immunoblot of NC‐HEK293‐exo and OV‐HEK293‐exo. (**d**) HUVECs were incubated with Dil‐labeled exosomes (red) for 24 hours before fluorescent and phase contrast images were captured, scale bar 50 μm. (**e**) The cellular levels of sFlt‐1 mRNA were measured in recipient HUVECs treated with NC‐HEK293‐exo (100 μg/mL) or OV‐HEK293‐exo (100 μg/mL) (*P < 0.05). (**f**) OV‐HEK293‐exo (100 μg/mL) inhibited HUVEC migration (*P < 0.05). (**g**) OV‐HEK293‐exo (containing 500 ng/mL sFlt‐1 protein) exhibited higher inhibition efficacy on pro‐angiogenesis of NCI‐H69‐exo in comparison with the same concentration of sFlt‐1 protein (*P < 0.05).

Therefore, we hypothesized that the pro‐angiogenesis of NCI‐H69‐exo can be rescued by adding exosomal sFlt‐1. We selected HEK293 cells, which possess high transfection efficiency, as a cell model to package sFlt‐1 to exosomes. We successfully established overexpression of sFlt‐1 in HEK293 cells using a method that was previously reported by Chang *et al*.^33^ The characteristics of exosomes isolated from overexpressed (OV) and negative control (NC) HEK293 cells showed no obvious differences (Fig 4a–d). To confirm that the engineered HEK293 cell‐secreted sFlt‐1 could be transferred to HUVECs by exosomes, we measured the sFlt‐1 mRNA levels in HUVECs treated with sFlt‐1‐loaded exosomes. An increase in cellular expression of sFlt‐1 was detected in recipient HUVECs treated with sFlt‐1‐loaded exosomes (Fig 4e). Consistent with the above results, sFlt‐1‐loaded exosomes inhibited HUVEC migration (Fig 4f). We have also demonstrated that sFlt‐1 protein or sFlt‐1‐enriched exosomes can inhibit the migration of HUVECs. Moreover, the addition of sFlt‐1‐loaded exosomes could reverse the pro‐angiogenesis of NCI‐H69‐exo. Furthermore, we found that sFlt‐1‐enriched exosomes exhibited higher inhibition efficacy on pro‐angiogenesis of NCI‐H69‐exo in comparison with the same concentration of sFlt‐1 protein (Fig 4g).

### SFlt‐1‐loaded exosomes suppress the tumor growth in vivo

**Figure 5:**
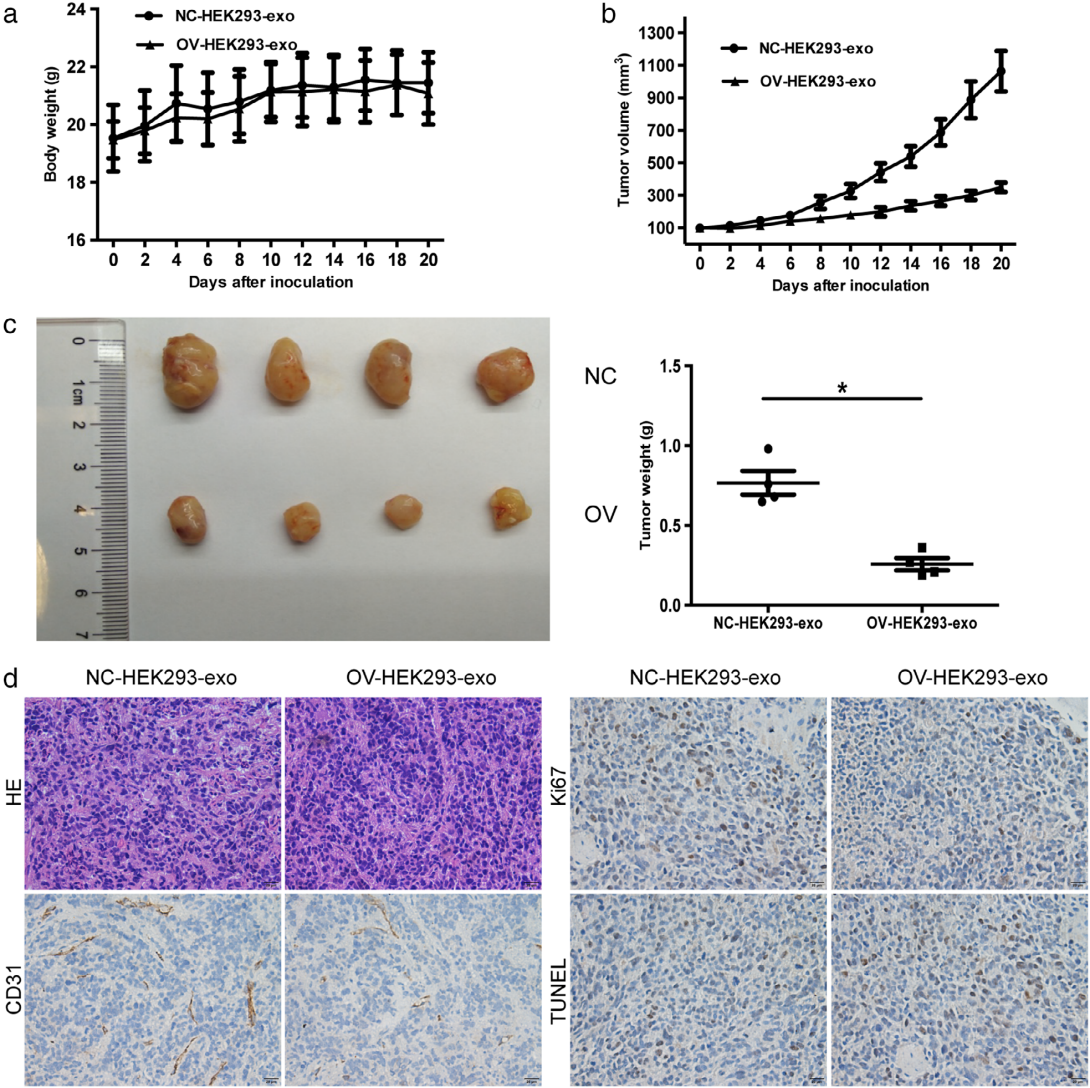
sFlt‐1‐loaded exosomes inhibit tumorigenesis in murine model. (**a**, **b**) Specific bodyweights and mean tumor volumes of subcutaneous xenografts NCI‐H69 after NC‐HEK293‐exo and OV‐HEK293‐exo treatment were shown at indicated time points (± SD). (**c**) Images of tumor samples in NC‐HEK293‐exo and OV‐HEK293‐exo treated xenografts (*P < 0.05). (**d**) HE staining, IHC staining for CD31, Ki67, TUNEL in NC‐HEK293‐exo and OV‐HEK293‐exo‐treated tumors. Scale bar 20 μm.

To further investigate the potential therapeutic effect of sFlt‐1‐loaded exosome intervention, we established subcutaneous xenografts in nude mice using the NCI‐H69 cell line. When the tumors averaged 100 mm^3^ in volume, the nude mice were treated by intratumoral administration with 100 μg of sFlt‐1‐loaded exosomes or control‐exosomes. None of the experimental nude mice had a weight loss of more than 10%, implying no significant signs of toxicity (Fig 5a). Intriguing, sFlt‐1‐loaded exosomes showed marked anti‐tumor activity by inhibiting the growth of NCI‐H69 tumor xenografts at the 20th day after injection (Fig 5b). After four weeks, mice treated with sFlt‐1‐enriched exosomes had a 66% decrease in average tumor weight as compared with control‐exosomes (Fig 5c). CD31 staining revealed that sFlt‐1‐loaded exosomes significantly reduced the vascular density of experimental mice (Fig 5d). We further measured tumor cell proliferation by Ki‐67 staining and tumor cell apoptosis by TUNEL staining. Figure 5d showed that sFlt‐1‐loaded exosomes markedly induced tumor apoptosis and inhibited tumor cell proliferation in mice. Based on the above results, we concluded that sFlt‐1‐loaded exosomes can be considered an effective therapeutic agent for SCLC.

## Discussion

In this study, we first explored the effect of exosomes derived from NCI‐H69 and BEAS‐2B cells on angiogenesis, to better understand the role of exosomes derived from SCLC cells. In comparison with BEAS‐2B‐exo, NCI‐H69‐exo contained less sFlt‐1 and significantly stimulated HUVEC migration. Meanwhile, sFlt‐1‐enriched exosomes reversed the pro‐angiogenesis of NCI‐H69‐exo on HUVECs. Furthermore, we demonstrated that sFlt‐1‐enriched exosomes suppressed tumor growth in nude mice, and was associated with increased tumor apoptosis, reduced microvessel density, and reduced tumor cell proliferation.

Under normal physiological conditions, angiogenesis is tightly regulated by a precise balance between angiogenic and anti‐angiogenic mediators. In addition, angiogenesis is comprised of several steps, including endothelium cell proliferation and migration.^38^ Exosomes are regarded as important mediators of the cancer cell microenvironment, communicating locally or at long distances to modulate recipient cells to promote tumor development.^15^, ^16^, ^17^ Zhou *et al*. found that the migration of endothelial cells was significantly enhanced by exosomes secreted by MDA‐MB‐231 metastatic breast cancer cells, but not by exosomes secreted by MCF‐10A noncancerous mammary epithelial cells.^16^ Moreover, Hsu *et al*. revealed that NSCLC‐derived exosomes can mediate intercellular communication to modulate tumor vasculature.^17^ In line with previous studies,^16^, ^17^ exosomes isolated from the NCI‐H69 cell line statistically promoted the migration of HUVECs. In this study, we discovered that SCLC‐secreted exosomes can be incorporated into HUVECs and stimulate the angiogenesis of HUVECs. Intriguingly, the sFlt‐1 level was significantly lower in serum from SCLC patients than healthy donors. Moreover, the protein expression of sFlt‐1 was also statistically lower in NCI‐H69‐exo than BEAS‐2B‐exo. It has been suggested that sFlt‐1 functions as a powerful anti‐angiogenic inhibitor by sequestering VEGF with high affinity.^22^, ^23^, ^24^ Accordingly, the angiogenesis function of NCI‐H69‐exo was significantly inhibited by treatment with sFlt‐1 protein. Our study indicates that the sFlt‐1 pathway is involved in the growth and development of SCLC.

Numerous preclinical studies have reported that the gene therapy system of sFlt‐1 can be applied as anti‐angiogenic therapy in nude mice transplanted with cancer cells.^25^, ^26^, ^27^, ^28^ Moreover, several preclinical studies have demonstrated that sFlt‐1 can suppress tumor growth and angiogenesis in NSCLC.^27^, ^28^ In addition, exosomes can facilitate the transport of their internal cargos by enhancing endocytosis, which liposomes and other synthetic drug nanoparticle carriers cannot do.^39^, ^40^ This suggests the idea of taking exosomes for drug delivery. Several studies have reported that exosomes can be engineered as delivery vehicles by transferring functional biomolecules to cancer cells.^29^, ^30^ Kamerkar *et al*. found that exosomes displayed a superb ability to deliver RNAi and inhibit pancreatic tumor growth.^41^

Since exosomes have emerged as an important tool for transferring bioactive materials for cell to cell communication, exosomes are gaining popularity as potential drug delivery vehicles. Therefore, we tested the potential role of exosomal sFlt‐1 in therapy for SCLC. In the current study, we successfully engineered HEK293 cells to secrete exosomes with plentiful sFlt‐1. These exosomes transferred high levels of sFlt‐1 protein to the recipient cells to reduce HUVE cell migration. In addition, we found that sFlt‐1‐enriched exosomes exhibited higher efficacy in inhibiting HUVEC migration compared to sFlt‐1 protein. As has been well established, exosomes have the ability to effectively deliver their cargo because of their transmembrane and membrane anchored proteins, which likely play important roles in enhancing endocytosis.^39^, ^40^ We performed an experiment in mice and demonstrated that sFlt‐1‐enriched exosomes can suppress tumor growth by reducing vascular density and increasing tumor cell apoptosis. These results show that sFlt‐1‐enriched exosomes may serve as a more efficient therapeutic agent than other vehicles for SCLC therapy.

In clinical practice, the therapeutic regimens for SCLC patients are limited. Clinical trials on anti‐angiogenic therapies have been disappointing, and no anti‐angiogenic drugs have been approved owing to lack of clinical efficacy. Angiogenetic inhibitors for treating SCLC include bevacizumab, sunitinib, and thalidomide. Bevacizumab is the most studied anti‐angiogenic agent in clinical trials of extensive‐stage SCLC. Recently, a phase III study showed that a combination of bevacizumab with standard platinum plus etoposide chemotherapy led to a meaningful improvement in progression‐free survival for the first‐line treatment of extensive‐disease SCLC with a tolerable toxicity profile.^20^ This implies that anti‐angiogenic therapy still shows promise for treating SCLC patients. Our preclinical study demonstrated that sFlt‐1‐enriched exosomes can significantly suppress the angiogenesis of SCLC tumors, and may be considered a potential anti‐angiogenic agent in the future. However, more work should be conducted to investigate the mechanisms of sFlt‐1‐loaded exosomes in treating SCLC.

In the present study, we report for the first time that exosomes from a SCLC cell line contain low levels of sFlt‐1 and significantly increased the migration of HUVECs. Next, we showed that sFlt‐1‐enriched exosomes can inhibit NCI‐H69‐exo‐induced HUVEC migration. In this preclinical study, we also demonstrated that exosomes enriched in sFlt‐1 have the potential to be effective therapeutic agents for SCLC.

## Disclosure

The authors declare that they have no competing interests.
